# Supplementary material for: Unveiling the Bioactive Potential of the Invasive Jellyfish Phyllorhiza punctata Through Integrative Transcriptomic and Proteomic Analyses
Source: Biomolecules. 2025 Aug 4;15(8):1121. doi: 10.3390/biom15081121 (PMC12383608; doi:10.3390/biom15081121)
Supplement: Supplementary file 1 [file biomolecules-15-01121-s001.zip › Suplementary Material/FileS3/PEPFOLD4/XP_065054023_5495/clusters.html]

 PEP-FOLD clusters


Download archive of all models  here   

|  |  |  |
| --- | --- | --- |
| # | file | sOPEP |
| # cluster 1 - Size: 3 |  |  |
|  | XP\_065054023\_5495-model1 | -18.1957 |
|  | XP\_065054023\_5495-model1.1 | -9.1574 |
|  | XP\_065054023\_5495-model1.2 | -7.92117 |
| # cluster 2 - Size: 3 |  |  |
|  | XP\_065054023\_5495-model2 | -16.2521 |
|  | XP\_065054023\_5495-model2.1 | -7.5519 |
|  | XP\_065054023\_5495-model2.2 | -7.12765 |
| # cluster 3 - Size: 1 |  |  |
|  | XP\_065054023\_5495-model3 | -16.1002 |
| # cluster 4 - Size: 1 |  |  |
|  | XP\_065054023\_5495-model4 | -15.6923 |
| # cluster 5 - Size: 2 |  |  |
|  | XP\_065054023\_5495-model5 | -15.404 |
|  | XP\_065054023\_5495-model5.1 | -9.25987 |
| # cluster 6 - Size: 1 |  |  |
|  | XP\_065054023\_5495-model6 | -15.1912 |
| # cluster 7 - Size: 2 |  |  |
|  | XP\_065054023\_5495-model7 | -14.7953 |
|  | XP\_065054023\_5495-model7.1 | -11.7718 |
| # cluster 8 - Size: 1 |  |  |
|  | XP\_065054023\_5495-model8 | -14.7533 |
| # cluster 9 - Size: 1 |  |  |
|  | XP\_065054023\_5495-model9 | -12.966 |
| # cluster 10 - Size: 1 |  |  |
|  | XP\_065054023\_5495-model10 | -12.2071 |
| # cluster 11 - Size: 1 |  |  |
|  | XP\_065054023\_5495-model11 | -11.2745 |
| # cluster 12 - Size: 1 |  |  |
|  | XP\_065054023\_5495-model12 | -11.1268 |
| # cluster 13 - Size: 1 |  |  |
|  | XP\_065054023\_5495-model13 | -10.825 |
| # cluster 14 - Size: 1 |  |  |
|  | XP\_065054023\_5495-model14 | -10.652 |
| # cluster 15 - Size: 1 |  |  |
|  | XP\_065054023\_5495-model15 | -10.2917 |
| # cluster 16 - Size: 1 |  |  |
|  | XP\_065054023\_5495-model16 | -9.19117 |
| # cluster 17 - Size: 2 |  |  |
|  | XP\_065054023\_5495-model17 | -9.12414 |
|  | XP\_065054023\_5495-model17.1 | -6.21234 |
| # cluster 18 - Size: 1 |  |  |
|  | XP\_065054023\_5495-model18 | -9.11593 |
| # cluster 19 - Size: 1 |  |  |
|  | XP\_065054023\_5495-model19 | -9.01901 |
| # cluster 20 - Size: 1 |  |  |
|  | XP\_065054023\_5495-model20 | -9.01344 |
| # cluster 21 - Size: 1 |  |  |
|  | XP\_065054023\_5495-model21 | -8.65738 |
| # cluster 22 - Size: 1 |  |  |
|  | XP\_065054023\_5495-model22 | -8.62725 |
| # cluster 23 - Size: 1 |  |  |
|  | XP\_065054023\_5495-model23 | -8.60749 |
| # cluster 24 - Size: 1 |  |  |
|  | XP\_065054023\_5495-model24 | -8.59962 |
| # cluster 25 - Size: 1 |  |  |
|  | XP\_065054023\_5495-model25 | -8.47489 |
| # cluster 26 - Size: 1 |  |  |
|  | XP\_065054023\_5495-model26 | -8.29738 |
| # cluster 27 - Size: 1 |  |  |
|  | XP\_065054023\_5495-model27 | -8.20756 |
| # cluster 28 - Size: 1 |  |  |
|  | XP\_065054023\_5495-model28 | -8.18795 |
| # cluster 29 - Size: 1 |  |  |
|  | XP\_065054023\_5495-model29 | -8.14645 |
| # cluster 30 - Size: 2 |  |  |
|  | XP\_065054023\_5495-model30 | -8.12052 |
|  | XP\_065054023\_5495-model30.1 | -7.76191 |
| # cluster 31 - Size: 1 |  |  |
|  | XP\_065054023\_5495-model31 | -8.03929 |
| # cluster 32 - Size: 1 |  |  |
|  | XP\_065054023\_5495-model32 | -7.84795 |
| # cluster 33 - Size: 2 |  |  |
|  | XP\_065054023\_5495-model33 | -7.7598 |
|  | XP\_065054023\_5495-model33.1 | -7.60894 |
| # cluster 34 - Size: 1 |  |  |
|  | XP\_065054023\_5495-model34 | -7.58894 |
| # cluster 35 - Size: 2 |  |  |
|  | XP\_065054023\_5495-model35 | -7.38139 |
|  | XP\_065054023\_5495-model35.1 | -5.53906 |
| # cluster 36 - Size: 1 |  |  |
|  | XP\_065054023\_5495-model36 | -7.32837 |
| # cluster 37 - Size: 1 |  |  |
|  | XP\_065054023\_5495-model37 | -7.31318 |
| # cluster 38 - Size: 1 |  |  |
|  | XP\_065054023\_5495-model38 | -7.31054 |
| # cluster 39 - Size: 1 |  |  |
|  | XP\_065054023\_5495-model39 | -7.29952 |
| # cluster 40 - Size: 1 |  |  |
|  | XP\_065054023\_5495-model40 | -7.16716 |
| # cluster 41 - Size: 2 |  |  |
|  | XP\_065054023\_5495-model41 | -7.15456 |
|  | XP\_065054023\_5495-model41.1 | -6.28505 |
| # cluster 42 - Size: 1 |  |  |
|  | XP\_065054023\_5495-model42 | -7.14852 |
| # cluster 43 - Size: 1 |  |  |
|  | XP\_065054023\_5495-model43 | -7.13813 |
| # cluster 44 - Size: 1 |  |  |
|  | XP\_065054023\_5495-model44 | -7.09375 |
| # cluster 45 - Size: 1 |  |  |
|  | XP\_065054023\_5495-model45 | -7.09047 |
| # cluster 46 - Size: 1 |  |  |
|  | XP\_065054023\_5495-model46 | -7.0271 |
| # cluster 47 - Size: 1 |  |  |
|  | XP\_065054023\_5495-model47 | -7.00444 |
| # cluster 48 - Size: 1 |  |  |
|  | XP\_065054023\_5495-model48 | -6.94389 |
| # cluster 49 - Size: 1 |  |  |
|  | XP\_065054023\_5495-model49 | -6.89973 |
| # cluster 50 - Size: 1 |  |  |
|  | XP\_065054023\_5495-model50 | -6.88347 |
| # cluster 51 - Size: 1 |  |  |
|  | XP\_065054023\_5495-model51 | -6.86175 |
| # cluster 52 - Size: 1 |  |  |
|  | XP\_065054023\_5495-model52 | -6.86031 |
| # cluster 53 - Size: 1 |  |  |
|  | XP\_065054023\_5495-model53 | -6.81649 |
| # cluster 54 - Size: 1 |  |  |
|  | XP\_065054023\_5495-model54 | -6.79581 |
| # cluster 55 - Size: 1 |  |  |
|  | XP\_065054023\_5495-model55 | -6.69662 |
| # cluster 56 - Size: 1 |  |  |
|  | XP\_065054023\_5495-model56 | -6.69346 |
| # cluster 57 - Size: 1 |  |  |
|  | XP\_065054023\_5495-model57 | -6.66376 |
| # cluster 58 - Size: 1 |  |  |
|  | XP\_065054023\_5495-model58 | -6.62621 |
| # cluster 59 - Size: 1 |  |  |
|  | XP\_065054023\_5495-model59 | -6.55927 |
| # cluster 60 - Size: 1 |  |  |
|  | XP\_065054023\_5495-model60 | -6.54156 |
| # cluster 61 - Size: 1 |  |  |
|  | XP\_065054023\_5495-model61 | -6.47557 |
| # cluster 62 - Size: 1 |  |  |
|  | XP\_065054023\_5495-model62 | -6.44809 |
| # cluster 63 - Size: 1 |  |  |
|  | XP\_065054023\_5495-model63 | -6.44574 |
| # cluster 64 - Size: 1 |  |  |
|  | XP\_065054023\_5495-model64 | -6.44059 |
| # cluster 65 - Size: 1 |  |  |
|  | XP\_065054023\_5495-model65 | -6.3864 |
| # cluster 66 - Size: 2 |  |  |
|  | XP\_065054023\_5495-model66 | -6.35762 |
|  | XP\_065054023\_5495-model66.1 | -5.93008 |
| # cluster 67 - Size: 1 |  |  |
|  | XP\_065054023\_5495-model67 | -6.34985 |
| # cluster 68 - Size: 1 |  |  |
|  | XP\_065054023\_5495-model68 | -6.33016 |
| # cluster 69 - Size: 3 |  |  |
|  | XP\_065054023\_5495-model69 | -6.32235 |
|  | XP\_065054023\_5495-model69.1 | -6.19877 |
|  | XP\_065054023\_5495-model69.2 | -5.60468 |
| # cluster 70 - Size: 1 |  |  |
|  | XP\_065054023\_5495-model70 | -6.24528 |
| # cluster 71 - Size: 1 |  |  |
|  | XP\_065054023\_5495-model71 | -6.24287 |
| # cluster 72 - Size: 1 |  |  |
|  | XP\_065054023\_5495-model72 | -6.24284 |
| # cluster 73 - Size: 1 |  |  |
|  | XP\_065054023\_5495-model73 | -6.21634 |
| # cluster 74 - Size: 1 |  |  |
|  | XP\_065054023\_5495-model74 | -6.20308 |
| # cluster 75 - Size: 1 |  |  |
|  | XP\_065054023\_5495-model75 | -6.16516 |
| # cluster 76 - Size: 1 |  |  |
|  | XP\_065054023\_5495-model76 | -5.9616 |
| # cluster 77 - Size: 1 |  |  |
|  | XP\_065054023\_5495-model77 | -5.96038 |
| # cluster 78 - Size: 1 |  |  |
|  | XP\_065054023\_5495-model78 | -5.88351 |
| # cluster 79 - Size: 1 |  |  |
|  | XP\_065054023\_5495-model79 | -5.85324 |
| # cluster 80 - Size: 1 |  |  |
|  | XP\_065054023\_5495-model80 | -5.76794 |
| # cluster 81 - Size: 1 |  |  |
|  | XP\_065054023\_5495-model81 | -5.67038 |
| # cluster 82 - Size: 1 |  |  |
|  | XP\_065054023\_5495-model82 | -5.64932 |
| # cluster 83 - Size: 1 |  |  |
|  | XP\_065054023\_5495-model83 | -5.52921 |
| # cluster 84 - Size: 1 |  |  |
|  | XP\_065054023\_5495-model84 | -5.47723 |
| # cluster 85 - Size: 1 |  |  |
|  | XP\_065054023\_5495-model85 | -5.42264 |
| # cluster 86 - Size: 1 |  |  |
|  | XP\_065054023\_5495-model86 | -5.41328 |
|  |  |  |
